# Silibinin strongly inhibits the growth kinetics of colon cancer stem cell-enriched spheroids by modulating interleukin 4/6-mediated survival signals

**DOI:** 10.18632/oncotarget.2068

**Published:** 2014-06-06

**Authors:** Sushil Kumar, Komal Raina, Chapla Agarwal, Rajesh Agarwal

**Affiliations:** ^1^ Department of Pharmaceutical Sciences, Skaggs School of Pharmacy and Pharmaceutical Sciences; ^2^ University of Colorado Cancer Center, University of Colorado Anschutz Medical Campus, Aurora, Colorado

**Keywords:** colorectal cancer, silibinin, cancer chemoprevention, cancer stem cells

## Abstract

Involvement of cancer stem cells (CSC) in initiation, progression, relapse, and therapy-resistance of colorectal cancer (CRC) warrants search for small molecules as ‘adjunct-therapy’ to target both colon CSC and bulk tumor population. Herein, we assessed the potential of silibinin to eradicate colon CSC together with associated molecular mechanisms. In studies examining how silibinin modulates dynamics of CSC spheroids in terms of its effect on kinetics of CSC spheroids generated in presence of mitogenic and interleukin (IL)-mediated signaling which provides an autocrine/paracrine amplification loop in CRC, silibinin strongly decreased colon CSC pool together with cell survival of bulk tumor cells. Silibinin effect on colon CSC was mediated *via* blocking of pro-tumorigenic signaling, notably IL-4/-6 signaling that affects CSC population. These silibinin effects were associated with decreased mRNA and protein levels of various CSC-associated transcription factors, signaling molecules and markers. Furthermore, 2D and 3D differentiation assays indicated formation of more differentiated clones by silibinin. These results highlight silibinin potential to interfere with kinetics of CSC pool by shifting CSC cell division to asymmetric type *via* targeting various signals associated with the survival and multiplication of colon CSC pool. Together, our findings further support clinical usefulness of silibinin in CRC intervention and therapy.

## INTRODUCTION

While colon resection is the treatment of choice for patients with localized colorectal cancer (CRC) [1], depending on the stage of the malignancy, adjuvant chemo/radio-therapy may still be required [1]. However, in spite of these treatment strategies, nearly 50% of CRC patients develop recurrent disease, and patients with advanced and metastatic CRC still succumb to death. The major reason for the failure of most of the treatment strategies is ascribed to the presence of cancer stem cells (CSC) in the tumor mass, which are essentially resistant to current therapeutic strategies, compared to bulk tumor cells [2-11]. As stem cells or their progenitors are the targets of transformation into CSC which are responsible for tumorigenesis, strategies that reduce CSC number, induce either apoptosis or differentiation with a loss of self-renewal capacity of CSC, or interfere with the pro-tumorigenic signals arising in the colon ‘niche’ that affects CSC population, represent a rational approach for CRC prevention and treatment [8, 11]. Thus, identification and development of drugs, especially non-toxic agents, which target these ‘tumor initiating cells’ might provide opportunities to intervene at the earliest [8, 11]; such an intervention at a late stage in cancer therapy would also be beneficial, as it would eradicate CSC pool, the presence of which results in cancer relapse [10]. Globally, several research efforts have reported the potential of a wide range of agents against CRC growth and progression [12]; however, in a broader perspective, the major limitation of these studies has been that they have not investigated the efficacy of these agents on colon CSC pool of the tumors. Despite the fact that last 5 years have seen a spurt in the anti-cancer and/or anti-CSC efficacy studies with natural agents, the efficacy of these agents towards colon CSC generation leading to colon tumorigenesis has not yet been well-defined.

In light of these perspectives, in this study, our focus was to examine and establish the efficacy of silibinin on colon CSC expansion, self-renewal and differentiation in the context of anti-CRC efficacy. Silibinin (a flavonolignan from milk thistle, *Silybum marianum*) is a natural agent with established strong efficacy (both preventive and therapeutic) against CRC xenografts in nude mice, azoxymethane-induced colon tumorigenesis in A/J mice and F344 rats, and spontaneous colon and small intestinal tumorigenesis in *APC*^min/+^ mice [13-19]. Furthermore, silibinin exerts strong anti-proliferative, pro-apoptotic and anti-inflammatory effects [12-22]; including strong potential to cause severe and irreparable damage to colon tumor cells by sustained interference in essential cellular processes, as seen in nuclear magnetic resonance spectroscopy-based metabolomics studies [23]. Previous studies delineating mechanisms of silibinin efficacy have also revealed that its effects were mainly due to the inhibition of Wnt/β-catenin pathway and associated transcriptional activity [13, 15, 17], which also plays an essential role in the transformation of stem cells as well as CRC development and progression [24-28]. Together, these results highlight the strong potential of silibinin in inhibiting the initiation and progression of CRC with associated mechanisms of action; however, these findings did not address whether silibinin also has the potential to affect colon CSC population. Since CSC are involved in CRC initiation and progression, relapse, and therapy-resistance, we rationalized that if silibinin targets CSC population in CRC, it would have strong translational preventive and therapeutic implications to control CRC clinically. Notably, silibinin is already in clinical trials in CRC patients, and completed studies have reported high silibinin bioavailability in the colonic tissue of CRC patients [29].

## RESULTS

### Silibinin exerts inhibitory effect on mitogen mediated-CSC enriched colonosphere formation

**Figure 1:**
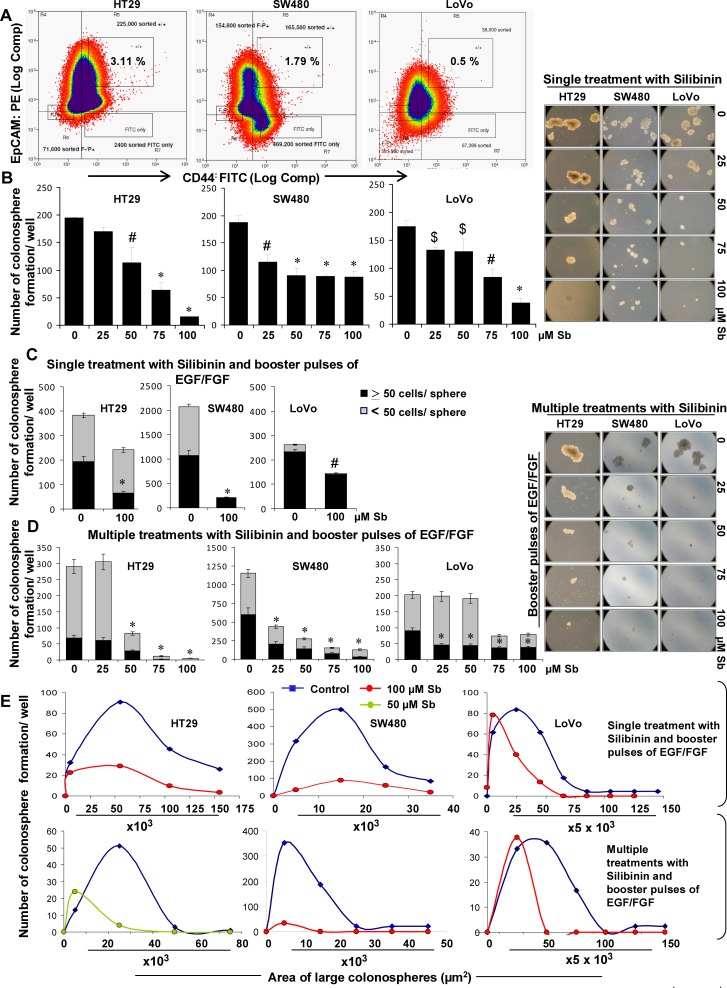
Effect of silibinin on the formation of CSC enriched colonosphere in HT29, SW480, and LoVo CRC cells A) FACS based identification of CSC in CRC cells based on CD44^+^EpCAM^high^ population. B) Effect of silibinin on colonosphere formation by CSC enriched CRC cells. Effect of silibinin, C) single treatment, D) multiple treatments on mitogen induced CSC enriched colonosphere formation. B& D-*left panels*) Representative photomicrographs (X100 magnification) of CSC enriched colonospheres depicting a decrease in their number and size by single and multiple treatments of silibinin. E) Comparative effect of single and multiple treatments of silibinin on the area of CSC enriched colonospheres after mitogen induction. Mitogen induction was done by addition of booster pulses of EGF (20ng/mL) and FGF (10ng/mL) every 72 hours. Silibinin (Sb) was added once during seeding (single treatment) or added every 72 h for multiple treatments. Area of colonospheres was measured using Zeiss Axioscope 2 microscope software (Carl Zeiss, Inc., Jena, Germany) and associated with number of colonospheres formed to determine the frequency of size range.$ *P*<0.05, # *P*<0.02; * *P*<0.001.

In CRC, recent studies have identified CD44^+^EpCAM^high^ cells as CSC, which retain key stem cell properties and drive tumor growth [3, 30, 31]. Typical properties of CSC are their ability to self-renew and their aberrant differentiation which drive tumorigenic events and contribute towards heterogeneity in tumor cell populations, respectively [4, 5, 7, 9, 10]. Accordingly, we first determined silibinin effect on self-renewal capacity of CSC population of CRC cell lines. For this, we isolated different cell populations (CD44^+^EpCAM^high^, CD44^+^EpCAM^low^, CD44^−^EpCAM^high^ and CD44^−^EpCAM^low^) from human CRC cell lines SW480, HT29 and LoVo (Fig.1A) and subjected them to sphere cluster formation assay to determine which of the isolated fractions was enriched in CSC population. Efficiency of colonosphere formation (data not shown) in different fractions was in the order of CD44^+^EpCAM^high^ >CD44^−^EpCAM^high^ >CD44^+^EpCAM^low^, with very few spheres in CD44^−^EpCAM^low^ fraction. The identified CSC enriched CD44^+^EpCAM^high^ sorted population was then subjected to sphere cluster formation assays in the absence or presence single treatment of silibinin (25-100μM), and % of floating spheroids (colonospheres) generated after 1-2 weeks were determined. Silibinin significantly decreased both number and size (Fig.1B) of colonospheres generated in all three CRC cell lines, which was also silibinin dose-dependent in HT29 and LoVo cell lines. While a decrease in number of colonospheres by silibinin highlights its effect on ‘tumor initiating cells’, the decrease in size (area/volume) shows its effect on bulk tumor/daughter cells.

Next, to mimic physiological influence of mitogens on colon CSC, sphere cluster formation assay was performed with additional booster pulses of mitogen growth factors [recombinant human epidermal growth factor (EGF) and fibroblast growth factor (FGF) EGF/FGF], which not only significantly increased the number of colonosphere formation per well but also additionally induced the formation of smaller spheres (<50 cells). In this assay, whereas a single treatment of silibinin was still able to significantly decrease the colonosphere formation, it lacked the potential to completely eradicate the sphere forming ability of CRC cells in the presence of additional mitogens (Fig.1C). Since CSC are inherently resistant to treatment modalities, a multiple treatment approach with silibinin was next employed to determine whether persistent exposure (though time bound) to silibinin (mimicking the real-time scenario where human cancer cells are continuously exposed to the treatment drugs during the course of therapy) could have a more significant effect on the sphere forming ability of these CRC cells. Indeed, multiple dosing of silibinin showed a more promising effect with a drastic decrease in colonosphere formation/well (Fig.1D) in presence of booster pulses of EGF/FGF. Furthermore, we calculated the area of individual colonospheres to determine how silibinin treatment affected the progenitor/ bulk CRC cells in the colonospheres (Fig. 1E). The frequency of colonospheres with larger area was higher in untreated controls, compared to silibinin groups in all three cell lines. It was also noted that in control LoVo colonospheres, the area of individual spheres was larger than in HT29 and SW480. Notably, the colonospheres exposed to multiple silibinin treatment had significantly lesser area, and the frequency of colonospheres with larger surface area was dramatically negligible in these groups (Fig. 1E). The data shown in case of HT29 cells with multiple silibinin treatments are at 50μM because 100μM showed almost complete inhibition (Fig. 1E).

### Silibinin alters the growth kinetics of CSC enriched colonospheres

**Figure 2:**
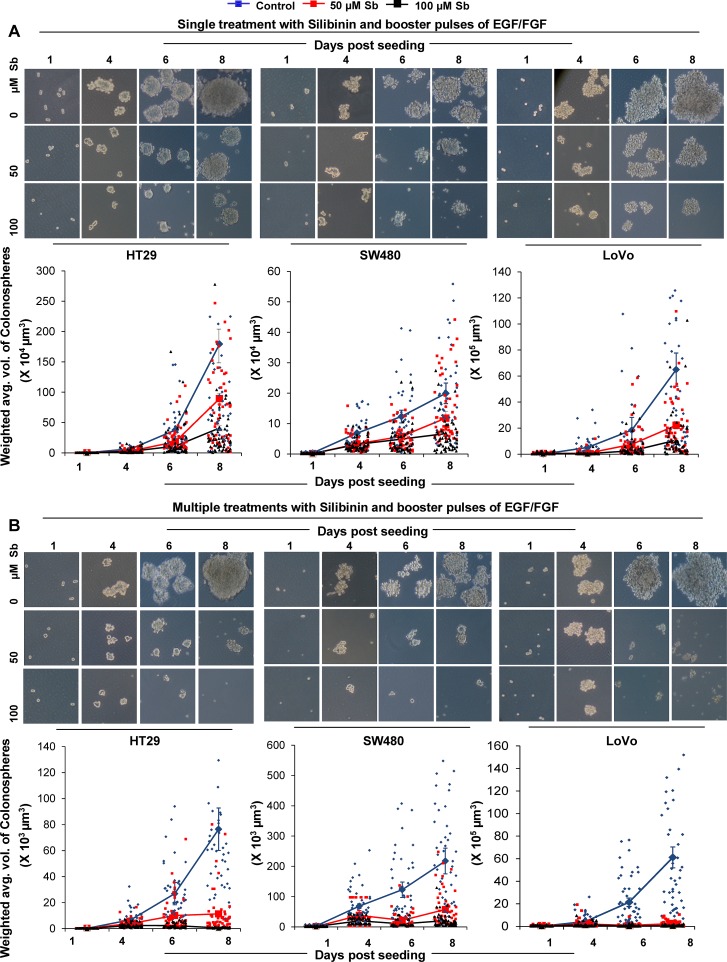
Effect of silibinin on the growth kinetics of CSC enriched colonospheres Effect of silibinin, A) single treatment, B) multiple treatments, on mitogen induced CSC enriched colonosphere formation as a function of time. Representative photomicrographs (X100 × 4 magnification) of CSC enriched colonospheres depicting sphere growth in absence and presence of single and multiple treatments of silibinin are shown in A& B-*upper panels*. Individual volume of the colonospheres was calculated by assuming that colonospheres were approximately spherical and that volume (V) =[4/3] πr^3^ which equals to [π/6] d^3^ where diameter ‘d’ is the average diameter (average of the longest diameter and the one perpendicular diameter to it) of colonospheres using Zeiss Axioscope 2 microscope software. Total volume was then associated with number of colonospheres formed to determine the weighted average volume of colonospheres.

Next we carried out studies to assess how silibinin modulates the dynamics of CSC spheroids (colonospheres), by examining its effect on the kinetics of CSC spheroids, generated from CRC cell lines. Comparative analysis of different cell lines in terms of sphere formation kinetics indicated that the CSC enriched colonospheres varied significantly in their growth kinetics (Fig. 2), measured as a function of time and related to the individual diameter and volume of the colonospheres. Effect of silibinin on the growth kinetics (Fig. 2A & B) of these CSC enriched colonospheres indicated a significant but differential modulatory effect on these properties across three CRC cell lines. While by day 4 of seeding, a significant decrease in the volume of colonospheres (calculated as weighted average volume) was seen in both single and multiple silibinin treatment groups, it was after 6 days that a dramatic decrease (91-99%) in the volume of colonospheres with multiple silibinin treatments was observed.

**Figure 3:**
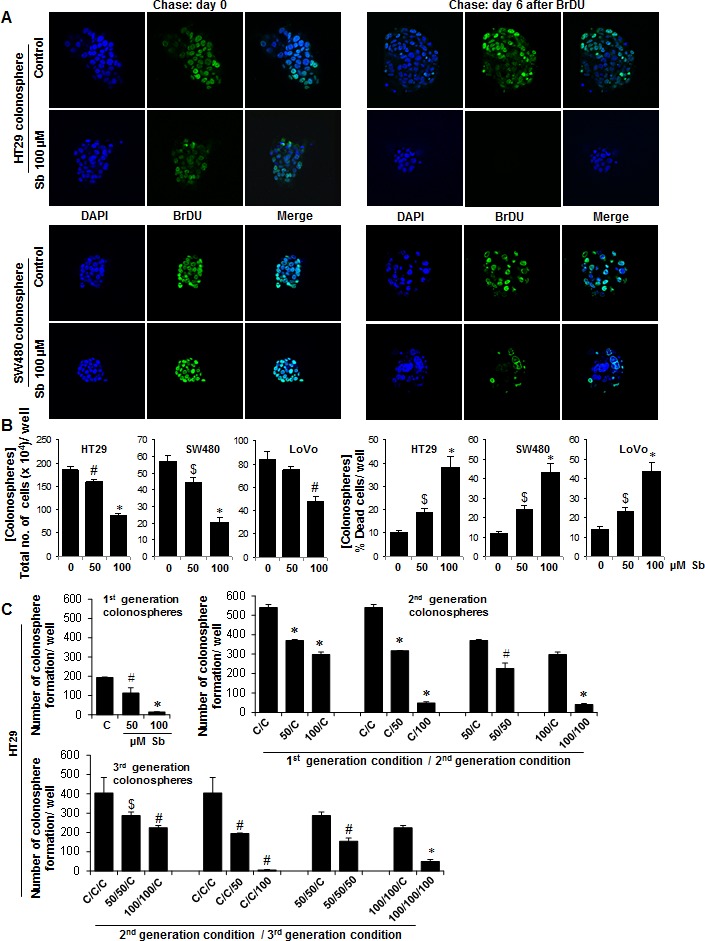
Effect of silibinin on the cycling properties of CSC and viability of CRC cells in the individual colonospheres A) Immunofluorescence staining showing a significant decrease in BrdU positive cells in colonospheres after silibinin treatment during chase experiments post BrdU exposure. Colonospheres were stained with anti-BrdU- FITC and counter stained with DAPI and representative photomicrographs (X600 magnification) of CSC enriched colonospheres are shown. B) Effect of silibinin on the viability of CRC cells in colonospheres. Colonospheres were dispersed as single cells by Accutase treatment, and Trypan blue dye exclusion assay was used to assess cell viability. C) Effect of silibinin on colonosphere formation in different generations. Equal number of viable cells from 1^st^ generation colonospheres (with or without silibinin treatment) were re-plated for 2^nd^ generation sphere cluster assay (with or without silibinin treatment) and this was process was repeated to generate 3^rd^ generation spheres. $ *P*<0.05, # *P*<0.02; * *P*<0.001.

For understanding in detail the mechanism of silibinin effect, we chose to work with colonospheres from only single silibinin treatment groups, as this had the advantage of large size colonospheres compared to those obtained after multiple treatments wherein sphere sizes were drastically reduced and not many spheres could be harvested at study end. To determine the effect on cycling properties of CSC which were enriched in the individual spheroids (Fig. 3A), colonospheres were subjected to pulse-chase experiments with BrdU. Immunofluorescence (IF) staining showed a significant decrease in BrdU positive cells after silibinin treatment during chase (3 and 6 days post BrdU exposure; only 6 day data is shown). Since CSC are slow dividing cells and retain BrdU for longer time periods, these results indicated that control colonospheres were highly enriched in CSC population compared to silibinin groups which showed less to negligible numbers of CSC population in colonospheres (Fig. 3A). Further assessment of growth inhibitory effect of silibinin in these colonospheres showed that silibinin caused a decrease in total cell numbers with a strong dose-dependent cell death in these colonospheres (Fig. 3B), accounting for 2-4 fold (P<0.05-P<0.001) increase in dead cell population per well (Fig. 3B). Next, equal number of live cells from the dissociated first generation colonospheres were seeded (3000 cells/well) for fresh sphere cluster formation assays in the presence or absence of silibinin, and allowed to form second generation colonospheres (Fig. 3C). This procedure, using the live cells from 2^nd^ generation colonospheres was repeated again to determine whether the effect of silibinin on the sphere forming ability of CSC persists in next generations (Fig. 3C). As shown in Figure 3C, the overall number of colonospheres increased in second generation compared to the first. However, colonospheres generated in subsequent generations by cells isolated from silibinin treated groups in previous generation were less compared to those generated from untreated controls, suggesting persistent effect of silibinin in next generations on the sphere forming ability of CSC. The representative data of HT-29 cells is shown, but similar effects were also observed in other two CRC cell lines namely SW480 and LoVo (data not shown).

### Silibinin exerts inhibitory effect on interleukin (IL)-mediated pro-tumorigenic signals in CSC enriched colonospheres

**Figure 4:**
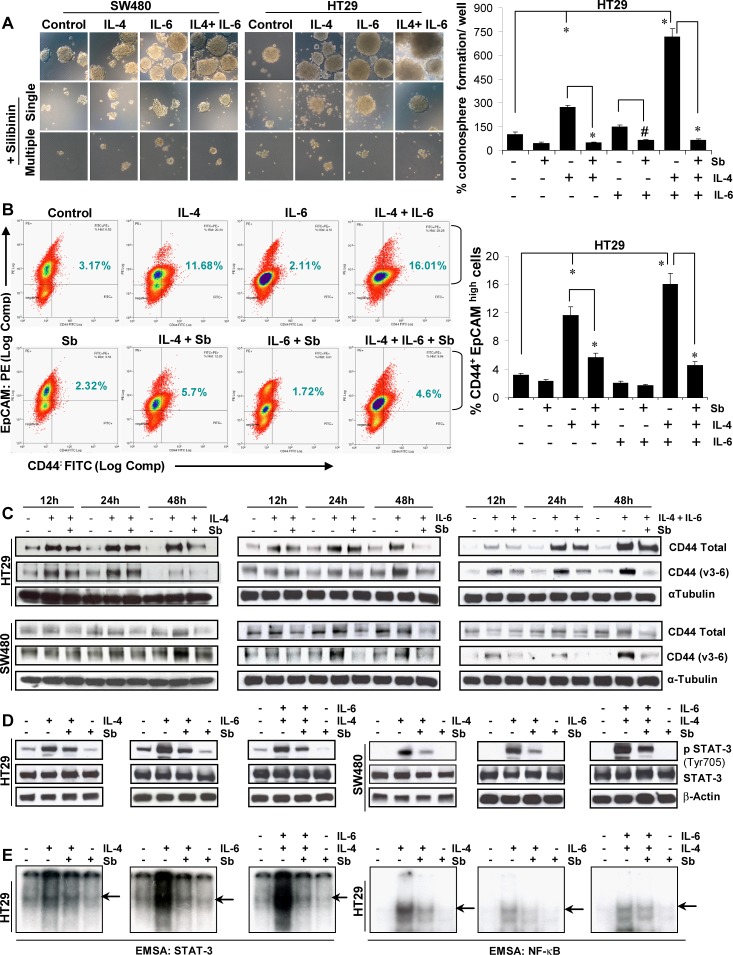
Effect of silibinin on the interleukin mediated pro-tumorigenic signals on CSC enriched colonospheres A) Effect of silibinin on size and number of colonospheres induced by IL-4 or IL-6 or their combination in sphere cluster formation assays. Representative photomicrographs (X100 × 3 magnification) of CSC enriched colonospheres are shown. Silibinin concentration: 100 μM (single treatment) and 50 μM (multiple treatments). B) Effect of silibinin (100 μM for 48h, under serum conditions) on the % of CD44^+^ EpCAM ^high^ cell population in CRC cells induced by IL-4 or IL-6 or their combination as detected by FACS. C) Time dependent effect of silibinin (100 μM Sb, under serum conditions) on IL-4 or IL-6 or their combination induced expression of CD44 and its variant form CD44 v3-v6 in CRC cells. D) Effect of silibinin on constitutive or IL-4 or IL-6 or their combination induced phosphorylation of STAT-3 (Tyr705) levels in CRC cells under serum starved conditions. Serum starved CRC cells were induced with IL, after 2 h treated with 100μM silibinin and then harvested after 9 h. E) Effect of silibinin on the transcription activity of STAT-3 and NF-κB in the nuclear lysates of CRC cells was analysed by EMSA. Representative autoradiograph gels, depicting the specific bands by arrows are shown. For authentication of bands, only labeled probe sample as well as unlabeled probe (or cold oligo) were also run together to determine band specificity (data not shown) Sb, silibinin; IL, interleukin. # *P*<0.02; * *P*<0.001.

Various studies have identified IL-4 or IL-6, produced by enterocytes and lamina propria myeloid cells, to a play critical role in both the survival and proliferation of pre-malignant intestinal epithelial cells as well as resistance of cancer stem cells to therapeutic treatments. Accordingly, next we chose to study whether silibinin effect on CSC is also mediated *via* blocking of signaling pathways mediated by these two interleukins. The sphere cluster assays were modified to mimic physiological influence of IL-4/-6 on CSC, and then silibinin effect on colonosphere formation was determined in their presence. As shown in Figure 4A, while IL-4 significantly increased the number of colonospheres, IL-6 only moderately increased their numbers; however, a most dramatic effect in sphere cluster assays (in terms of both number and size of colonospheres) was observed when a combination of IL-4 and IL-6 was used (Fig. 4A, *left panel*). Quantitatively, ~7 fold (p<0.001) increase in HT-29 colonosphere formation in presence of IL-4 and IL-6 combination was seen (Fig. 4A, *right panel*), with similar observations in other CRC cell lines (data not shown). Overall, silibinin completely inhibited the effects of IL-4 and IL-6 and their combination on colonospheres formation (Fig. 4A). Furthermore, silibinin was also able to significantly decrease the IL-4 and/or IL-6 mediated increase in CD44^+^EpCAM^high^ positive CRC cells (Fig. 4B). In the mechanistic studies to delineate how silibinin reversed the pro-tumorigenic effects of IL-4/-6 and their combination on colon CSC, monolayer culture experiments showed that treatment of CRC cell lines with 100μM silibinin was able to significantly reduce IL-4/-6 induced expression of both total CD44 and its variant CD44v3-6 (highly expressed in CRC tumors) in a time-dependent manner (Fig. 4C). IL-4 and/or −6 are critical NF-κB-dependent pro-tumorigenic cytokines, which also stimulate survival and proliferation *via* oncogenic transcription factor STAT-3 [32-39]. Accordingly, subsequent studies were carried out to determine if silibinin had any effect on these signals. Results showed that indeed silibinin inhibits constitutive as well as IL-4/-6 induced activation of transcription factor STAT-3 in terms of its Tyr705 phosphorylation in CRC cells (Fig. 4D). Qualitative electrophoretic mobility shift assay (EMSA) was next performed to further confirm the effect of silibinin on IL-induced activation of both STAT-3 and NFκB transcription factors. As evident in Figure 4E, the IL-4 and/or IL-6 induced DNA binding activity of these molecules was significantly reduced by silibinin. The representative data are shown only in HT-29 cells but similar effects were also observed in SW480 cells (data not shown). The validity of gel-shift bands for STAT-3 and NFκB was established as reported earlier [22, 40, 41] (data not shown).

### Silibinin alters the gene levels of CSC associated-transcription factors, signaling molecules, and markers in CSC enriched colonospheres

**Figure 5:**
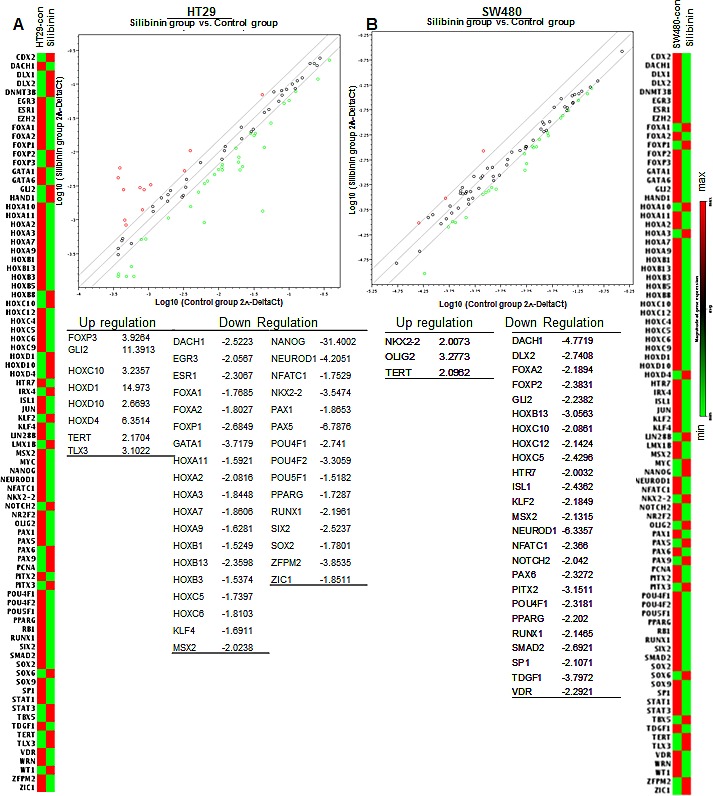
Effect of silibinin on stem cell associated transcription factors in mitogen mediated CSC enriched colonospheres Effect of silibinin on, A) HT29 and B) SW480 colonospheres. Treatments and other details are provided in Materials and methods. For RT^2^qPCR analysis of human stem cell transcription factors, RT^2^ Profiler™ PCR Array (Qiagen) was used.

**Figure 6:**
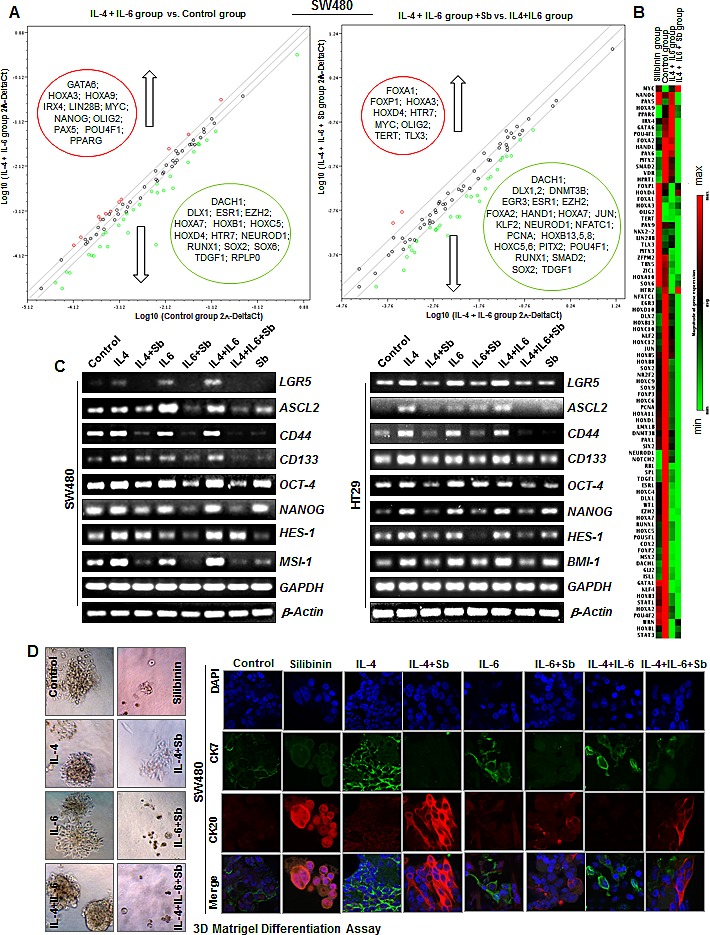
Effect of Silibinin on mRNA levels of CSC associated-transcription factors, signaling molecules, and markers in interleukin mediated CSC enriched colonospheres A) Effect of Silibinin on stem cell associated transcription factors in IL-4 and IL-6 combination mediated CSC enriched SW480 colonospheres as determined by RT^2^qPCR using Human stem cell transcription factor RT^2^ Profiler ™ PCR Array (Qiagen). B) Representative heat map showing relative changes in genes estimated by RT^2^qPCR. C) Effect of Silibinin on *LGR5, ASCL2, CD133*, *CD44, OCT-4, NANOG, MSI-1, HES-1, and BMI-1* mRNA levels as determined by semi-quantitative RT-PCR analysis. Treatments and other details are provided in Materials and methods. D) Effect of silibinin on 3D differentiation of CSC enriched colonospheres. For 3D differentiation, colonospheres were allowed to differentiate in GF-reduced Matrigel in regular culture media containing 10% FBS. Representative phase contrast photomicrographs (X100 × 2.3 magnification) of CSC enriched colonospheres are shown in D*-left panel* while IF staining (X 600 magnification using confocal microscopy) of 3D differentiated colonospheres with CK20 and CK7 with DAPI as nuclear stain are shown in D*-right panel.*

To examine whether silibinin efficacy against colon CSC involves altered expression of various stem cell transcription factors, we utilized human stem cell transcription factor RT^2^qPCR array to analyze the expression of ~84 genes associated with stem cells in the colonospheres of CRC cell lines (Fig. 5). Results indicated that silibinin causes an alteration in the expression of various CSC associated transcription factors both in the absence (Fig.5) and presence of IL-4 and IL-6 combination (Fig. 6); though, the effects were differential across cell lines (*Supplementary Figures 1 & 2*) and varied depending upon the presence of cytokine during colonosphere generation (Fig. 6A & B). Notably, in HT29 cells (Fig. 5A), silibinin alone caused a significant decrease in the expression of NANOG (~31 folds), *NEUROD1, PAX5, PPARG, SOX-2, RUNX1, EGR3, DACH1 and GATA1* gene levels; while it increased *GLI2, TERT, TLX3*, and *HOXD-1, −4, −10* levels. Consistent with its effect in HT29 cells, silibinin also decreased the level of *NANOG* gene by ~13 folds in LoVo cells (*Supplementary Figure 1*); other genes that were significantly decreased were *SOX-2*, *SOX-9, EGR3 and FOXP1; while NKX2-2, SOX-6, WT1 and ZIC1* gene levels were increased. In SW480 cells, a ~4-6 fold decreased was observed in *DACH1* and *NEUROD1* gene levels; while more than 2 folds decrease was observed in *DLX2*, *NFATC1, PPARG, RUNX1, TDGF1, NOTCH2, HOXB13* and *MSX2* gene levels by silibinin alone (Fig. 5B). Similar to other cell lines, the gene levels of *TERT* and *NKX2-2* were increased by silibinin in SW480 cells (Fig. 5B). In additional studies where IL-4 + IL-6 combination was used as booster in SW480 cell lines, the genes that were significantly affected by the addition of silibinin were: *DACHI, DLX1, HOXC5, HOXB13, NEUROD1, SOX-2, TDGF1* and *EGR3* which were down regulated and *HTR7*, *TERT* and *TLX3* which were up regulated (Fig. 6 and *Supplementary Figure 2*). Furthermore, silibinin also significantly decreased IL-4 + IL-6 mediated increase in *POU4F1, NANOG, PPARG* and *PAX5* gene levels. Additional comparative analysis of modified gene levels across three different CRC cell lines (HT-29, SW480 and LoVo) indicated that silibinin significantly and consistently mediates its effect by down regulation of *NANOG, NEUROD1, SOX-2, DACH1, EGR3, POU4F1* and *PPARG* genes, while at the same time, up regulating *TERT* levels. Of these results, the effects on *NANOG* and *SOX-2* genes are of utmost significance for the current study as these genes are implicated in CSC pool expansion [42-48].

To further confirm the changes in the expression of stem cell associated transcription factors by silibinin, we next performed semi-quantitative RT-PCR analysis on the selected colon CSC associated genes, and found that IL-4 or IL-6 alone or their combination induces the expression of various colon CSC associated genes, which was significantly decreased by silibinin (Fig. 6C). Specifically, silibinin down regulated the mRNA levels of IL-4 and/or IL-6 induced: a) CSC-associated markers and transcription factors, notably, *LGR5, ASCL2, CD44, CD133*, *OCT-4, NANOG, MSI-1 and BMI-1*, and b) other regulatory molecule, such as *HES-1*, the transcriptional target of NOTCH 1 signaling. These results are important as transcriptional activity of these genes controls the fate of CSCs, and is required to induce a stem-like phenotype and to activate an anti-apoptotic program in human cancer cells [42-55]. Importantly, a strong expression of these molecules is also associated with poor prognosis and an advanced stage of the disease in various malignancies including CRC [42, 43, 47, 49, 50, 56].

### Silibinin induces differentiation in CSC enriched colonospheres

Colonospheres were also subjected to 2D and 3D differentiation assays in presence of serum containing media to determine whether silibinin had the potential to induce more differentiated clones in CSC enriched colonospheres [31, 57-59]. Phase contrast microscopy in 2D assay showed that the colonospheres had started to adhere to bottom of the plate and show the signs of differentiation quite early in silibinin treated groups; however, control and the IL-4 and/or IL-6 induced colonospheres displayed delayed differentiation (data not shown). Notably, at later stages, control colonospheres spread at a faster rate compared to silibinin groups where relatively more damaged/dead cells were apparent (data not shown). The 3D differentiation assay further corroborated the above findings in 2D (Fig. 6D, *left panel*). At the end of the experiment, differentiated cells were subjected to IF staining for CK20 and CK7 and observed under confocal microscopy, where a higher ratio of CK20/CK7 was observed [31, 57-59] indicating increased differentiation in silibinin treated groups including those in the presence of IL-4 and/or IL-6 treatments, compared to non-silibinin groups (Fig. 6D, *right panel*).

### Silibinin decreases the protein expression of CSC associated-transcription factors, signaling molecules, and markers in CSC enriched colonospheres

**Figure 7:**
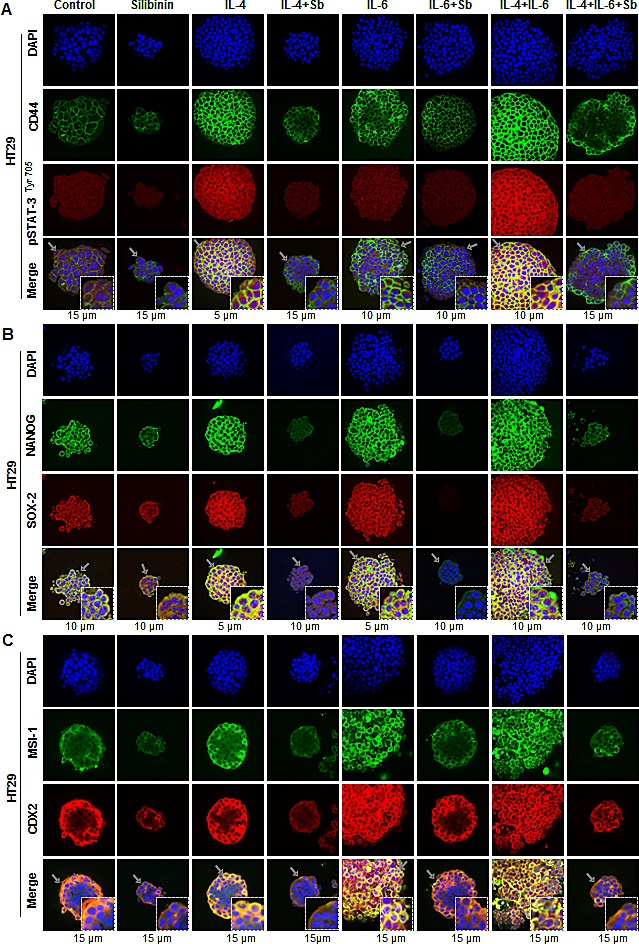
Effect of Silibinin on the protein expression of CSC associated-transcription factors, signaling molecules, and markers in interleukin mediated CSC enriched colonospheres Effect of silibinin on protein expression of, A) CD44 and pSTAT-3 ^Tyr705^; B) NANOG and SOX-2; and C) MSI-1 and CDX2 levels in HT29 colonospheres in the presence of IL-4 or IL-6 or their combination. Generated colonospheres were harvested, immobilized in matrigel, and stained with respective fluorescent antibodies. Z stack analysis using confocal microscopy was performed as detailed in ‘Materials and Methods’ section and representative scans (X 600) with individual scan depth of specific colonospheres are shown. Inserts in merged images show magnified sections (digital magnification: 3x) of colonospheres

Based on the data showing that silibinin modulates the mRNA expression of colon CSC associated transcription factors and regulatory molecules in colonospheres, we next assessed the expression of their effector protein molecules in the colonospheres (Fig. 7). The Z stack analysis of colonospheres was performed, which revealed that how protein expression of these essential colon CSC-associated molecules has changed by IL-4 and/or IL-6 and how silibinin modulate them. Similar to data showing that IL-induced expression of CD44 was mediated by increased Tyr phosphorylation of Stat-3, we found that indeed IL-4 and/or IL-6 increases the expression of both CD44 and pSTAT-3 ^Tyr705^ in the colonospheres (Fig. 7A). CD44 expression was increased more towards the peripheral zone of the colonospheres after IL induction compared to untreated colonospheres (*Supplementary Figure 3*). Increased cytosolic and moderate pSTAT-3 ^Tyr705^ nuclear staining was observed with IL induction compared to controls in both central and peripheral regions of the colonospheres (*Supplementary Figure 3*). A combination of both IL-4 and IL-6 drastically increased the pSTAT-3 ^Tyr705^ nuclear expression which was effectively decreased to basal levels by silibinin (Fig.7A), suggesting that indeed IL-4 and IL-6 combination generated more pro-tumorigenic signals that influenced the growth of CSC enriched spheroids, the effects of which were successfully negated by silibinin treatment. Next, evaluation of NANOG and SOX-2 pattern in the colonospheres yielded that IL-4 had the potential to more significantly induce their expression (both cytosolic and nuclear) compared to IL-6, which was effectively reduced by silibinin to levels even lesser than controls (Fig.7B). Similar effects were observed with staining for MSI-1 and CDX2 (Fig.7C); their induced expressions were also reduced by silibinin. Representative z scans showing staining pattern (at various depths) of these molecules in colonospheres is shown in *Supplementary Figure 3*.

## DISCUSSION

CSC can undergo any one of the following types of mitotic events: a) symmetric self-renewal, with probability r_1_, in which CSC generates two daughter cells with CSC characteristics of parent cell [CSC→CSC +CSC]; b) asymmetric self-renewal, with probability r_2_, in which CSC generates one CSC and one 1^st^ generation progenitor cell [CSC→ CSC +P_1_]; and c) symmetric commitment, with probability r_3_, in which CSC generates two 1^st^ generation progenitor cells [CSC→ P_1_ + P_1_]. Under normalization condition r_1_ + r_2_ + r_3_ = 1; mathematical modeling predicts that ρ_S_, the overall rate of CSC division depends upon the frequency at which each CSC can undergo any one of these specific cell division events [60-64]. Progenitor cells, the transit amplifying cells downstream of CSC, undergo only a limited number of cell division events before they terminally differentiate. The net effect of these divisions of progenitor cells is an increase in the number of mature cells. Thus, the rate of tumor/colonosphere size increase also depends upon cell populations generated; the symmetric CSC division drives/maintains tumor growth more aggressively than the asymmetric CSC division [61, 64]. The mathematical modeling of CSC division has generated different hypotheses which predict different strategies to target CSC and bulk tumor cells [60, 61, 64]. One of the universally accepted hypotheses is that the drugs which target bulk tumor cells can result in significant tumor shrinkage, but this strategy results in increased fraction of CSC population. The remaining CSC populations self-renew resulting in CSC enriched tumors which have an aggressive phenotype and are more drug resistant. Based on this hypothesis, one of the strategies to combat tumor cells efficiently, is a combined treatment approach that targets both CSC and non-CSC population leading to significant tumor shrinkage as well as decreased drug resistance and tumor relapse [60]. However, another hypothesis based on above mathematical model is that terminally differentiated bulk tumor cells negatively affect, by feed back mechanisms, the probability of self-renewal and proliferation rates of CSC; their presence causes CSC to proceed towards asymmetric cell division at a slower proliferation rate. Thus, conventional drug therapy that targets only progenitor and terminally differentiated bulk tumor cells causes a shift from asymmetric to symmetric cell division of CSC with increased proliferation rate [61]. Accordingly, this alternative hypothesis puts forth a treatment strategy that does not support targeting of both CSC and bulk tumor cells as it would indirectly remove the negative feed-back on the CSC self-renewal; it in fact proposes targeting of only CSC for efficient anti-tumor efficacy and prevention of tumor relapse [61]. Based on above perspectives, one common theme is to target CSC population, and thus the non-toxic agents with anti-CSC potential could be a rational approach for CRC prevention and treatment. We selected silibinin as one such investigational drug, because our recent studies have shown that it causes strong anti-CRC efficacy, and its ability to spare normal colon cells but initiate a severe programmed cell death in CRC cells [23].

In the present study, employing CSC enriched population of CRC cell lines, we have shown that silibinin decreases the percentage of CSC and that it has an inhibitory effect on both number and size of colonospheres. Since formation of colonospheres is a measure of stemness, our results provide the evidence that silibinin has the potential to target the self-renewal of CSC as well as bulk tumor cells in CRC. This combined efficacy of silibinin against both cell types obviously did not compromise its efficacy in inhibiting colonosphere growth rate and also did not increase the chances for tumor relapse as evident from persistent efficacy of silibinin in next generation colonospheres; these findings are in contrast to above discussed alternative hypothesis. Mechanistically, the observed effects of silibinin could be related to its ability to transform/differentiate CD44^+^ population into a CD44^−^ phenotype, as evidenced by decreased mRNA/protein levels of CD44 and the fact that silibinin increases differentiation of CRC cells in colonospheres. Such transformation of phenotype could make these cells more sensitive to silibinin, thus supporting the fact that its multiple treatments were more effective in reducing both the number and size of the colonospheres. Since mathematical modeling has also predicted that symmetrical self-renewal of CSC is mainly responsible for CRC growth and progression, there is a possibility that silibinin, due to its dual efficacy against both cell types, is also shifting CSC cell division to asymmetric type.

Inflammatory milieu of the CSC niche is another important component where its components regulate the growth of both CSC and progenitor cell populations [33, 34]. The inflammatory signals such as cytokines arising in CSC niche, due to the presence of inflammatory cells, also network with other regulatory pathways to influence the expansion of both cell types. Activation of IL-4/-6 mediated signaling cascade in colon enterocytes and myeloid cells in the lamina propria regulates the cell survival/proliferation of pre-malignant intestinal epithelial cells. Additionally, cytokine IL-4 is also implicated in increased resistance of CSC to chemotherapy agents, and blocking of IL-4 signaling is shown to increase the sensitivity of CSC to apoptosis leading to increased efficacy of cytotoxic therapies [32-34, 37, 38, 65, 66]. Taken together, targeting IL-4/IL-6 signaling could be an additional strategy to control colon CSC pool expansion for the prevention and treatment of CRC. In this regard, our results show that silibinin strongly decreases the pro-tumorigenic effects of cytokines by reducing both the percentage of colon CSC and colonosphere formation, which were mediated *via* blocking of IL-4/-6 signaling by silibinin in different CRC cell lines. Notably, silibinin caused a strong decrease in IL-4/-6 induced activation of STAT-3 and NF-κB transcriptional activity, which was also associated with a decrease in mRNA/protein levels of various CSC regulatory molecules, and CSC-associated markers, transcription factors, and stemness genes, such as, *OCT-4, NANOG, SOX-2, etc*. Furthermore, the fact that silibinin affects the key regulatory signals originating from the stem cell niche, such as Wnt-/β-catenin/FGF/Notch family proteins that play a critical role in the growth and survival of CSC during colon carcinogenesis [27, 55, 67, 68], is another important observation emphasizing the potential of silibinin to interfere with the signals in stem cell ‘niche’ that support colon CSC survival and pool expansion.

## CONCLUSION

In the present study, the anti-CRC efficacy of silibinin has been evaluated extensively with central emphasis on targeting both colon CSC/bulk tumor cells. Our data provide sufficient evidence that silibinin has dual efficacy against both bulk CRC cells and colon CSC, wherein it interferes with kinetics of CSC pool expansion, shifting CSC cell division to asymmetric type and also inducing their differentiation *via* targeting various regulatory signals associated with the survival and multiplication of colon CSC pool. These findings form a rational basis for future *in vivo* studies to examine and establish silibinin efficacy on targeting colon CSC. Considering that silibinin consumption is safe [69, 70] and that it has remarkable efficacy against CRC in *in vivo* rodent models [13-17, 19], with high bioavailability in colon tissue of CRC patients [29, 70], the present study further supports the use of silibinin clinically, both as a CRC prevention strategy and as an ‘adjunct therapy’ to be effective in cases where current anti-cancer modalities fail to cause complete eradication of CRC due to the lack of their potential to target colon CSC.

## MATERIALS AND METHODS

### Reagents and Cell Culture

Silibinin was from Sigma-Aldrich and dissolved in DMSO. Antibodies used were: CD44 total/ variant v3-6, α-Tubulin, MSI-1 (Santa Cruz Biotechnology); SOX-2, pSTAT-3^Tyr705^ and total STAT-3 (Cell Signaling); NANOG, CK20, CK7, CDX2, BrdU-FITC (Abcam) and β-Actin (Sigma-Aldrich). CD44-FITC and EpCAM-PE antibodies were from BD Biosciences. TexasRed or Alexa Flour 488/594 conjugated secondary antibodies, EGF and FGF were from Invitrogen while IL-4 and IL-6 were from Millipore. Consensus sequences of STAT-3 and NF-κB oligonucleotides and the gel shift assay system were from Santa Cruz Biotechnology and Promega Corp, respectively. HT29, SW480 and LoVo CRC cells (from ATCC) were grown under standard adherent culture conditions in DMEM, RPMI-1640, and F-12 media (Gibco), respectively, containing 10% FBS and 1% penicillin-streptomycin. Cells at 60% confluency, either serum starved (SS) [for 36 h (HT29) and 24 h (SW480), for detecting pSTAT3 protein levels and EMSA] or in complete media [(containing 10% FBS), for detecting CD44 protein levels], were stimulated with interleukin IL-4 or IL-6 alone (20 ng/mL each) or their combination, and after 2h treated with 100 μM silibinin and harvested at required intervals. Whole-cell extracts for western blotting (WB) and nuclear lysates for EMSA were prepared, protein concentrations determined, EMSA (gel shift and competitions assays) and WB performed as described previously [22, 40, 41].

### Sphere cluster formation assays

At log phase, adherent CRC cells were trypsin digested, dissociated into single cells, stained with CD44-FITC and EpCAM-PE antibodies (BD Pharmingen), and then subjected to cell sorting by FACS using Flow Cytometry Shared Resources of the UCCC. The isolated cell populations, dilutions ranging from1×10^4^ to 300 cells/ well were then subjected to sphere cluster formation assays. Briefly, sorted cells, carefully dispersed as single cells, were cultured in stem cell specific serum free media (2mL) in an ultra-low attachment six well plates (Costar) for 10-12 days. This defined media [DMEM/F-12(1:1 ratio) supplemented with 1% penicillin-streptomycin, B27 and N2 supplements (all from Gibco), and growth factors [recombinant human epidermal growth factor (EGF) and fibroblast growth factor (FGF), both from Invitrogen] supports the growth of stem cell fraction of cells only, and with time, cells proliferate to form floating single cell cloned spheres, known as colonospheres in colon and/or CRC cells. After seeding, cells were observed on daily basis to ensure that spheres were forming as a result of cell multiplication and not due to adherence of nearby cells. Since 3000 cells per well of CD44^+^EpCAM^high^ cells generated optimum number of colonospheres (CSC enriched fraction), these were used in all future assays.

Depending upon treatment conditions, sphere cluster assays were performed with FACS sorted CD44^+^EpCAM^high^ cells in the absence or presence of extra booster doses of growth factors [EGF, 20ng/mL and FGF, 10ng/mL] and/or interleukins [IL-4 or IL-6 alone or their combination (20ng/mL each)] with or without silibinin. Briefly, fresh media (500μL) and booster additives (growth factors and/or IL) were added every 72h. Silibinin was added initially after 6 hrs of seeding, and then added every 72h in case of multiple treatment approach. Colonospheres with ≥ 50 cells were scored as large (true colonospheres), while colonospheres <50 but >15 cells were considered as small spheres. Cellular viability was measured using Trypan blue dye exclusion after dissociating spheres with Accutase for 15 min at 37°C. For second and third generation of colonospheres, equal number (3000 cells/well) of these live cells from the dissociated colonospheres were re-seeded in the presence (50-100 μM) or absence of silibinin, and allowed to form next generation colonospheres (Fig. 3C). Briefly, live cells from silibinin treated colonospheres in the first generation either served as controls or were treated with silibinin in subsequent generations, and the sphere forming ability was compared with that of live cells of untreated control colonospheres which were also re-cycled in next generations.

### Immunofluorescence (IF) staining of colonospheres

Generated colonospheres were harvested and immobilized in matrigel for staining convenience. Briefly, 8 well chamber glass slides (BD Falcon) were coated with thin layer of matrigel [40μl/well of growth factor (GF) reduced matrigel, BD Bioscences]. Approximately, about 100 pre-washed colonospheres in phosphate buffered saline (PBS) were mixed (1:1 ratio) with 4% matrigel in PBS and 400 μl layered on top of the solidified basement matrigel coated chamber slide. These immobilized colonospheres were formalin fixed, permeabilized and incubated with respective primary antibodies followed by fluorochrome-conjugated secondary antibodies (Invitrogen). Briefly, the immobilized colonospheres were fixed in 3.75% buffered formalin for 30min at room temperature (RT) followed by gentle rinsing in 500 μl PBS, 3 times for 5min each. These were next permeabilized in PBST (PBS + 0.3% Triton X-100) for 2h with gentle shaking; thereafter, they were blocked for 60min in CAS block buffer (Invitrogen, 1:1 in PBS). Colonospheres were then incubated with respective primary antibodies in dilution buffer (1% BSA in PBST) overnight at RT in humidified chamber and subsequently washed three times in 0.1% Triton X-100 containing PBST. This was followed by incubation in dark for 1h with fluorochrome-conjugated secondary antibodies followed by one wash with 0.1% Triton X-100 containing PBST and two washes with high salt PBS. Samples were mounted with Prolong® Gold Antifade Reagent /DAPI and covered with a cover slip. Stained colonospheres images were captured using a Nikon D-Eclipse C1 confocal microscope (Nikon) and analyzed using EZ-C1 Free viewer software. Z stacking was performed by overall scanning of colonosphere in depth and then a reference point was selected in the middle from where scans of 5μM interval were taken in both directions till last visible point. For comparisons, averaged, interval scans that best represent the highest fluorescence intensity/signal were used.

### BrdU labeling of colonospheres

Following specific treatments, colonospheres were pulsed with BrdU labeling reagent (Invitrogen) at day 5 of sphere cluster assay for 48h and then BrDU was removed by changing media, and cells were chased for six days (chase: 0, 3, and 6 days post BrdU exposure) in BrdU free stem cell specific serum free media to continue the sphere cluster assay. At intervals of 0, 3 and 6 days post BrdU exposure the harvested colonospheres were transferred/ immobilized in matrigel on chambered slide for IF staining as described above, with slight modification. Briefly, after formalin fixation and washing, samples were subjected to acid treatment (1.5M HCl) for 30min at room temperature followed by blocking as described above. FITC-conjugated BrdU antibody was used to stain the BrdU positive cells and processed, as described in IF staining of colonospheres for visualization under confocal microscopy.

### Two/ three dimensional (2D/ 3D) differentiation assay of colonospheres

Generated colonospheres were either allowed to differentiate under 2D adherent conditions, as monolayer, in regular cell culture media (10% FBS), or cultured under 3D conditions. Briefly, for 3D differentiation, harvested colonospheres were embedded in GF-reduced matrigel as described above, with slight modifications; colonospheres were suspended in regular cell culture media (10% FBS) with 2% matrigel and then layered on basement matrigel. The entire setup was incubated at 37°C for 1 week, and media was replaced every 48h. Both the defined conditions under serum, allowed the cells to gradually migrate from colonospheres and differentiate. At the end of the experiment, differentiated cells were subjected to IF staining for differentiation markers CK 20 and CK 7.

### RT-PCR and RT2qPCR analysis of RNA extracted from colonospheres

Total RNA from accutase dispersed colonospheres was isolated by Trizol^R^ method, its integrity checked, genomic DNA eliminated, and first-strand cDNA prepared using RT^−^PCR first strand kit (Qiagen). Specific primers (*Supplementary Table 1*) were obtained from Sigma and used for semi-quantitative RT-PCR analysis. The PCR mix comprised 2X RedExtract-N-Amp PCR Ready Mix (Sigma) 12.5 μl, 10 pmol each forward and reverse primers (Sigma), and 2 μl cDNA in a total volume of 25μl. Thermal parameters for the amplification were: initial denaturation for 5min at 94°C; 30 cycles of denaturation for 30s at 94°C, annealing for 30s at 54°C and extension for 30 s at 72°C; and final extension for 5min at 72°C. The amplified products were subjected to electrophoresis on a 2.5% agarose gel in TAE buffer [40mM Tris/acetate (pH8.0), 1mM EDTA] containing 0.5μg/ml ethidium bromide (Sigma) and visualized on a gel documentation unit (Bio-Rad). For RT^2^qPCR analysis of human stem cell transcription factors, RT^2^ Profiler™ PCR Array (Qiagen) was employed. Total RNA was extracted from colonospheres by Trizol extraction method as above, reverse transcription was performed using 2-3 μg of RNA and First strand system for RT-PCR (Qiagen), and subjected to RT^2^qPCR analysis using Human stem cell transcription factor RT^2^ Profiler ™ PCR Array (Qiagen). A two-step cycling protocol on ABI 7500 cycler was used involving denaturation for 10 min at 95°C followed by 40 cycles of 15sec at 95°C and 1min at 60°C. The relative quantification of gene expression between control and silibinin treated samples was achieved by normalization against endogenous *GAPDH* and *β-Actin* using the ΔΔC_T_ method of quantification and the data was analyzed using the software provided by the manufacturer.

### Statistical Analysis

Difference between treatment groups was determined by one-way ANOVA or un-paired two-tailed Student's *t-*test using Sigma stat 2.03 software. Two-sided P value of 0.05 was considered significant.

## SUPPLEMENTARY MATERIAL, FIGURES AND TABLE


