# Supplementary material for: Silibinin strongly inhibits the growth kinetics of colon cancer stem cell-enriched spheroids by modulating interleukin 4/6-mediated survival signals
Source: Oncotarget. 2014 Jun 6;5(13):4972–89. doi: 10.18632/oncotarget.2068 (PMC4148115; doi:10.18632/oncotarget.2068)
Supplement: Supplementary file 1 [file oncotarget-05-4972-s001.pdf]

Silibinin strongly inhibits the growth kinetics of colon cancer stem cell-enriched spheroids by modulating interleukin 4/6-mediated survival signals

Supplementary Material

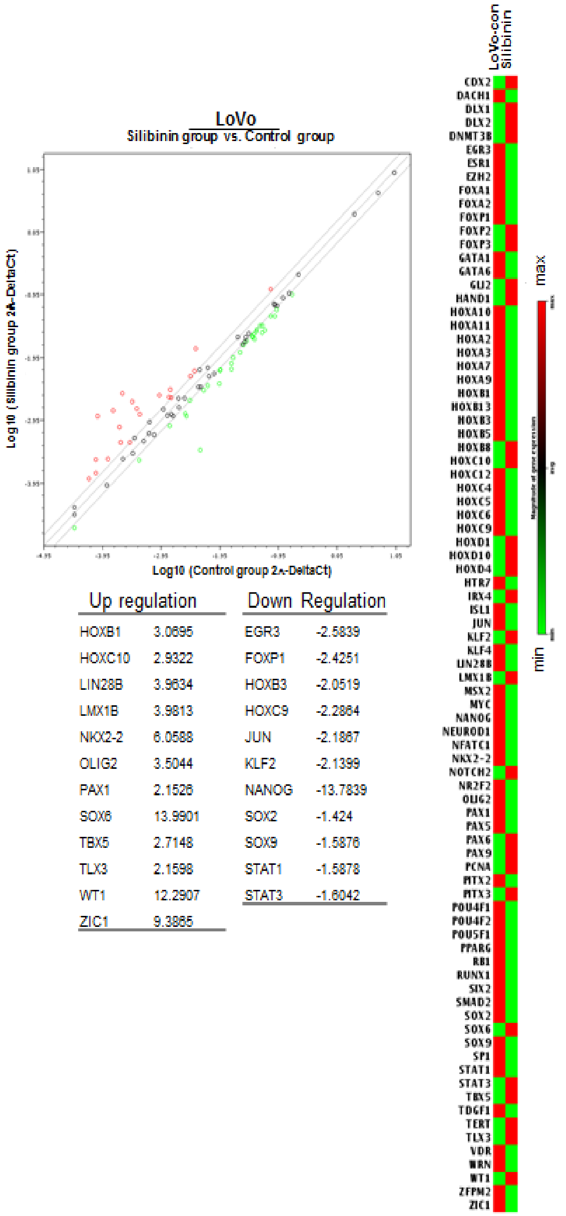

Up regulation		Down Regulation	
HOXB1	3.0695	EGR3	-2.5839
HOXC10	2.9322	FOXP1	-2.4251
LIN28B	3.9634	HOXB3	-2.0519
LMX1B	3.9813	HOXC9	-2.2864
NKX2-2	6.0588	JUN	-2.1867
OLIG2	3.5044	KLF2	-2.1399
PAX1	2.1526	NANOG	-13.7839
SOX6	13.9901	SOX2	-1.424
TBX5	2.7148	SOX9	-1.5876
TLX3	2.1598	STAT1	-1.5878
WT1	12.2907	STAT3	-1.6042
ZIC1	9.3865		

Supplementary Figure 1: Effect of silibinin on stem cell associated transcription factors in mitogen mediated CSC enriched colonospheres of LoVo cells. Total RNA was extracted from colonospheres by Trizol extraction method, reverse transcription was performed using 2-3 µg of RNA and first strand system for RT-PCR (Qiagen), and subjected to RT2qPCR analysis using Human stem cell transcription factor RT2 Profiler TM PCR Array (Qiagen).

A

IL-4+IL-6 vs. Control			
Gene	Up Regulation	Gene	Down Regulation
GATA6	1.7151	CDX2	-1.9794
HOXA3	1.5375	DACH1	-35.9239
HOXA9	1.9627	DLX1	-4.7502
IRX4	1.7599	ESR1	-1.9916
LIN28B	1.6683	EZH2	-2.0327
MYC	1.6218	FOXP2	-2.8604
NANOG	1.6519	GATA1	-3.4505
OLIG2	1.6245	GLI2	-2.0136
PAX5	2.0943	HOXA7	-2.1468
POU4F1	1.6456	HOXB1	-3.0898
PPARG	2.0718	HOXB13	-1.5205
		HOXB8	-1.6502
		HOXC4	-4.4991
		HOXC5	-3.1737
		HOXC6	-1.5977
		HOXD4	-2.4084
		HTR7	-2.4445
		ISL1	-1.9306
		KLF4	-1.5951
		LMX1B	-1.5173
		MSX2	-2.5308
		NEUROD1	-1.6483
		PITX3	-2.0034
		RUNX1	-2.0248
		SOX2	-1.8701
		SOX6	-2.0987
		STAT3	-1.7626
		TDGF1	-2.6327
		WT1	-1.8467
		ZFPM2	-1.6803
		RPLP0	-2.2761
		HGDC	-1.6473

B

IL4+IL6+Sb vs. IL-4+IL-6			
Gene	Up Regulation	Gene	Down Regulation
FOXA1	1.6697	CDX2	-1.7181
FOXP1	1.6723	DACH1	-152.3095
HOXA3	2.0787	DLX1	-7.9946
HOXD4	2.2463	DLX2	-3.316
HTR7	1.9428	DNMT3B	-3.3246
MYC	2.3042	EGR3	-3.317
OLIG2	2.3149	ESR1	-2.1392
TERT	1.6571	EZH2	-1.542
TLX3	1.6444	FOXA2	-4.0914
		FOXP2	-2.5464
		GATA1	-2.5269
		GLI2	-2.1568
		HAND1	-1.8132
		HOXA7	-1.7856
		HOXB13	-4.347
		HOXB3	-1.8104
		HOXB5	-2.4487
		HOXB8	-2.4345
		HOXC10	-2.221
		HOXC12	-2.4553
		HOXC4	-5.5565
		HOXC5	-13.6745
		HOXC6	-3.3397
		HOXC9	-2.2772
		HOXD1	-1.6887
		HOXD10	-1.8392
		ISL1	-2.248
		JUN	-2.555
		KLF2	-4.2741
		LMX1B	-2.7216
		MSX2	-2.4176
		NEUROD1	-4.1009
		NFATC1	-3.8039
		NR2F2	-1.5094
		PCNA	-1.6814
		PITX2	-6.9035
		PITX3	-2.3565
		POU4F1	-1.672
		RUNX1	-3.3485
		SIX2	-1.6184
		SMAD2	-3.6579
		SOX2	-4.6815
		SP1	-1.5408
		TDGF1	-10.1399
		VDR	-1.6598
		WT1	-1.8114
		ZFPM2	-1.7164
		RPLP0	-1.6036

Supplementary Figure 2: Effect of silibinin on stem cell associated transcription factors in IL-4 and IL-6 combination mediated CSC enriched colonospheres of SW480 cells. Total RNA was extracted from colonospheres by Trizol extraction method, reverse transcription was performed using 2-3 μg of RNA and first strand system for RT-PCR (Qiagen), and subjected to RT2qPCR analysis using Human stem cell transcription factor RT2 ProfilerTM PCR Array (Qiagen). List of genes modified by, A) IL-4 + IL-6 combination, and B) IL-4 + IL-6 combination in presence of silibinin.

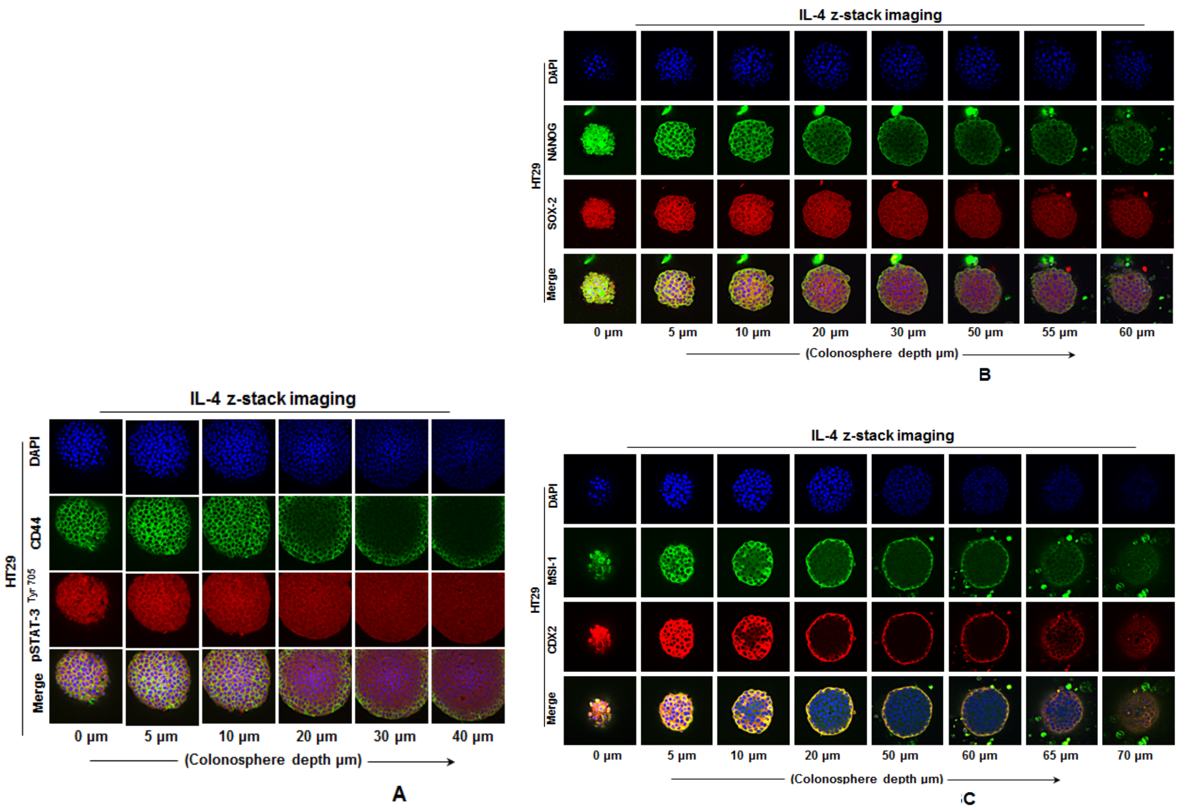

Supplementary Figure 3: Z stack images of A) CD44 and pSTAT-3Tyr 705; B) NANOG and SOX-2; C) MSI-1 and CDX2 staining in IL-4 mediated CSC enriched HT29 colonospheres. Stained colonospheres images (X 600 magnification) were captured using a Nikon D-Eclipse C1 confocal microscope (Nikon) and analyzed using EZ-C1 Free viewer software. Z stacking was performed by overall scanning of colonosphere in depth and then selection of a reference point in middle from where scans of 5µM interval were taken in both directions till last visible point.

Supplementary Table 1: Primer sequence of genes used for semi-quantitative analysis by RT-PCR.

Gene	Primer (5'-3')
LGR5 (F)	GGTGACAACAGCAGTATGGACGA
LGR5 (R)	GAAGGTGAACACTGCACTGAATGAA
ASCL2 (F)	CGTGAAGCTGGTGAACTTGG
ASCL2 (R)	GGATGTACTCCACGGCTGAG
CD44 (F)	AGATCAGTCACAGACCTGCC
CD44 (R)	GCAAACTGCAAGAATCAAAGCC
CD133 (F)	CAGAGTACAACGCCAAACCA
CD133 (R)	AAATCACGATGAGGGTCAGC
OCT-4 (F)	TCCCATGCATTCAAACTGAGG
OCT-4 (R)	CCAAAAACCCTGGCACAAACT
NANOG (F)	TGGACACTGGCTGAATCCTTC
NANOG (R)	CGTTGATTAGGCTCCAACCAT
HES-1 (F)	TCAACACGACACCGGATAAA
HES-1 (R)	CCGCGAGCTATCTTTCTTCA
BMI-1 (F)	AATGTCTTTTCCGCCCGCT
BMI-1 (R)	ACCCTCCACAAAGCACACACAT
MSI-1 (F)	GAGGGTTCGGGTTTGTCACG
MSI-1 (R)	GGCGACATCACCTCCTTTGG
GAPDH (F)	CCCCTGGCCAAGGTCATCCA
GAPDH (R)	ACAGCCTTGGCAGCGCCAGT
**β-ACTIN** (F)	GAGCGCGGCTACAGCTT
**β-ACTIN** (R)	TCCTTAATGTCACGCACGATTT
